# Understanding interferometry for micro-cantilever displacement detection

**DOI:** 10.3762/bjnano.7.76

**Published:** 2016-06-10

**Authors:** Alexander von Schmidsfeld, Tobias Nörenberg, Matthias Temmen, Michael Reichling

**Affiliations:** 1 Fachbereich Physik, Universität Osnabrück, Barbarastraße 7, 49076 Osnabrück, Germany

**Keywords:** displacement noise spectral density, interferometer, non-contact atomic force microscope (NC-AFM), opto-mechanic effects

## Abstract

Interferometric displacement detection in a cantilever-based non-contact atomic force microscope (NC-AFM) operated in ultra-high vacuum is demonstrated for the Michelson and Fabry–Pérot modes of operation. Each mode is addressed by appropriately adjusting the distance between the fiber end delivering and collecting light and a highly reflective micro-cantilever, both together forming the interferometric cavity. For a precise measurement of the cantilever displacement, the relative positioning of fiber and cantilever is of critical importance. We describe a systematic approach for accurate alignment as well as the implications of deficient fiber–cantilever configurations. In the Fabry–Pérot regime, the displacement noise spectral density strongly decreases with decreasing distance between the fiber-end and the cantilever, yielding a noise floor of 24 fm/Hz^0.5^ under optimum conditions.

## Introduction

A common method for measuring the displacement of a micro-cantilever or another micro-mechanical device is interferometric displacement detection. The most basic interferometer setup is the Michelson interferometer using two mirrors for the superposition of two light beams [1–2]. A related interferometric setup based on multi-beam interference in an optical cavity is the Fabry-Pérot interferometer typically used in form of an etalon in spectroscopy, lasers, and optical telecommunication [3] for precise wavelength selection within a certain free spectral range [4]. The Fabry–Pérot interferometer is characterized by the finesse 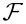, defined as the ratio between the spectral selectivity and the free spectral range [5]. Both types of interferometers are suitable for precisely detecting small movements of one of the involved mirrors [6]. The high precision and sensitivity of calibrated position measurement makes the interferometer a suitable system for displacement detection in a cantilever based non-contact atomic force microscope (NC-AFM) [7]. In contrast to a classical interferometer, the setup commonly involving a fiber end and a cantilever is characterized by a significant beam divergence and a small mirror area. Such a system is susceptible to misalignment resulting in increased optical loss in the cavity and a strongly reduced signal-to-noise performance. In a previous publication, we have shown that using the bare, cleaved fiber end allows one to change the characteristics of the interferometer from Fabry–Pérot to Michelson interference by adjusting the distance between the fiber end and the cantilever [8].

The micro-cantilever used for force detection in an interferometry-based NC-AFM is a lightweight oscillating mirror, which is part of an optical cavity and, therefore, its movement can be affected by forces originating from the radiation pressure acting on the cantilever [9]. Under conditions of Fabry–Pérot interference, this yields an optical spring effect, i.e., an effective cantilever stiffness that is increased or lowered depending on the slope of the interference fringe [10–11]. In previous work, we have shown that the variation of the distance between the fiber end and the cantilever allows for a control of the opto-mechanical interaction between the cavity light field and the cantilever [8]. The type and quality of interference can be straightforwardly assessed by measuring the Fabry–Pérot enhancement factor 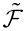, which is defined as

[1]
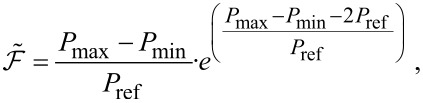


In case of negligible optical loss at very small distances *d*, 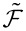 is related to the cavity finesse 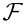 by

[2]
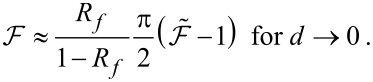


Here, we discuss the beams involved in generating the interference pattern in a typical cantilever setup and describe a systematic approach for optimum adjustment based on the externally measurable optical signals. This comprises fine-tuning the fiber–cantilever distance such that the resting position of the cantilever is exactly at the steepest point of the cavity response function, yielding maximum signal response for any given deflection.

To further optimize the signal, we introduce a method for positioning the fiber precisely in the optimal lateral position and examine the importance of this positioning. Finally, we investigate the impact of the interferometric signal on the effective modal spring constant 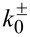 and the modal Q-factor 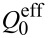 of the cantilever, as well as on the noise floor 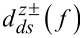 of the deflection measurement.

## Experimental

**Figure 1:**
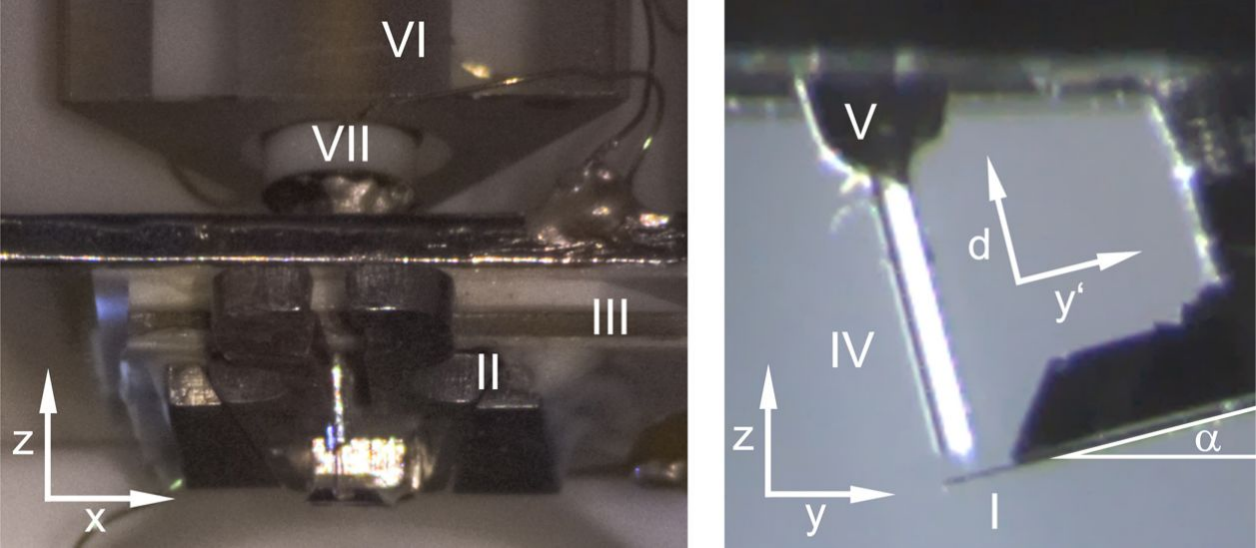
Details of the NC-AFM measuring head in a front and side view showing the interferometric setup with the cantilever, I, mounted on a dove-tail cantilever holder, II, inserted in a holding mechanism on the piezo stage, III. The fiber, IV, is mounted in the ferrule, V, glued in the tube piezo, VII, used for fine-positioning and scanning the fiber. The tube-scanner piezo is embedded in the sapphire prism, VI, which is part of the coarse-approach mechanism. The side view shows the fiber retracted by 200 μm from the cantilever.

Experiments are performed with a NC-AFM body operated in an ultra-high vacuum (UHV) environment with a base pressure of 3 × 10^−11^ mbar. The main components of the interferometer are shown in Figure 1, while all further details of the NC-AFM setup are described in [12]. The cantilever, I, with its support chip is mounted on a dove-tail cantilever holder, II, clamped into position inside two side braces on a piezo stage, III, facilitating cantilever excitation. The cantilever holder has an angle of α = 15° with respect to the horizontally aligned sample surface to make sure that the tip at the cantilever end approaches the sample surface first. For the studies reported here, the sample is, however, always retracted so that the cantilever displacement is affected only by the cavity light field but not by any tip–sample interaction.

The optical fiber, IV, is glued in a ferrule, V, which is bent by 15° with respect to the vertical axis to match the cantilever angle. The fiber end is coarse-approached from the top with a piezoelectric actuator moving the triangular sapphire prism, VI, along the *z*-axis towards the cantilever in steps of 0.4 μm. The actuator is electrically driven by a PMC100 stepper control system (RHK Technology Inc., Troy(MI) USA). The tube piezo, VII, inside the prism allows for positioning the fiber end in *x*-, *y*- and *z*-directions and, specifically, for scanning laterally over an area of 20 μm × 20 μm. A SPM1000 scan controller (RHK Technology Inc., Troy(MI) USA) is used to drive the scanning tube piezo, while a custom-built, low-noise power supply is used to position the fiber exactly at the optimal position.

Most experiments are performed with three aluminium-coated silicon micro-cantilevers taken from one batch (type NCLR, NanoWorld AG, Neuchâtel, Switzerland) further on referred to as cantilever 1, 2 and 3. They have a reflectivity of *R*_c_ ≈ 90% for light with a wavelength of λ = 782 nm, a length of 220 μm, a width of 40 μm and a thickness of 7 μm. Cantilever 3, which exhibits the best results, is used for dynamic measurements involving oscillation at its eigenfrequency of *f*_0_ = 164,999 Hz at a measuring head temperature of *T* = 29.3 °C. Some measurements are performed with cantilever 4 (type NCHR, NanoWorld AG, Neuchâtel, Switzerland) having similar properties as the others, but a length of 125 μm, a width of 30 μm and a thickness of 4 μm.

**Figure 2:**
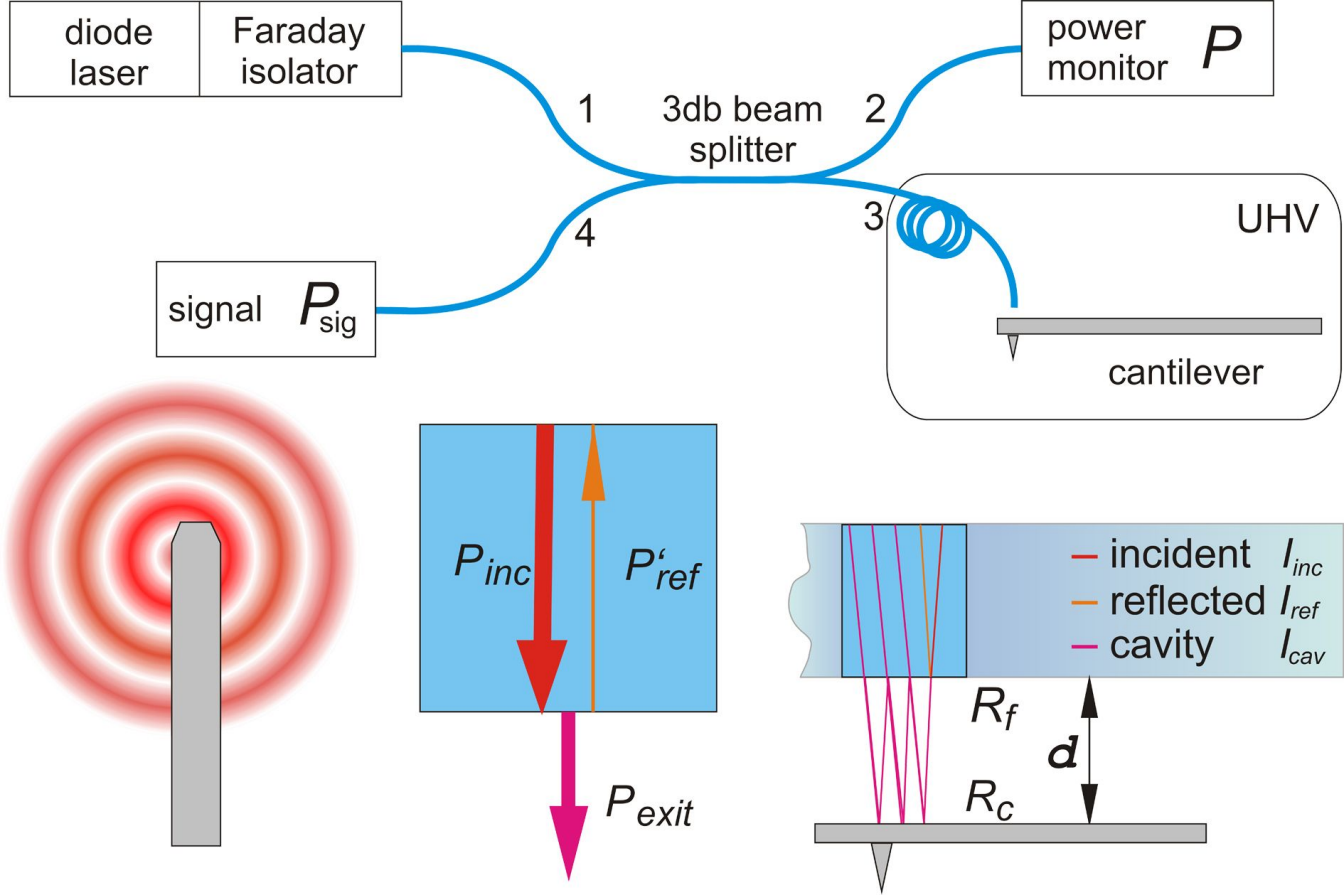
Schematic representation of the interferometer setup, signal path and cavity parameters. Signal power *P*_sig_ and power monitor *P* are either measured with a power meter or processed with a balanced photo detector for low-noise detection of dynamic signals.

The optical setup shown in Figure 2 consist of a stabilized laser light source (type 48TA-1-42037, Schäfter + Kirchhoff GmbH, Hamburg, Germany) operated at a wavelength of λ = 782 nm with the output power being optimized for low-noise operation. The power of the light coupled into the interferometer is optically adjusted by a variable absorber. A single-mode optical fiber with a core diameter of 4.0 μm (type Hi780, Corning Inc., Corning, New York, USA) optimized for transmission of light of the utilized wavelength is used to transmit the light from the source to the components of the interferometer setup. The fiber end placed above the cantilever is cleaved with great care to achieve a high interface reflectivity *R_f_*. The reflectivity is determined by procedures outlined below and we regularly obtain *R_f_* values higher than 3.5%. The best value obtained is *R_f_* = (3.9 ± 0.3)%, which is –within experimental error– identical to the maximum possible value of 3.84% determined by the diffraction index of the core material of the fiber (*n* = 1.48 at 800 nm according to the data sheet). No coating to increase the reflectivity has been applied to the cleaved end, resulting in a strongly asymmetric optical cavity that allows us to tune the interferometer from Fabry–Pérot to Michelson characteristics [8].

The laser is decoupled from the interferometric detection system through a Faraday isolator feeding the light into port 1 of the 3 dB coupler that divides the beam in two parts with identical light power *P* exiting at ports 2 and 3. The 3 dB splitting is confirmed by a measurement with an optical power meter (type TQ8210, Advantest, Tokyo, Japan). Port 2 is used as the power monitor while light from port 3 is directed onto the cantilever via the optical fiber guiding the light into the UHV chamber. To determine the optical loss in the feed line of the fiber occurring after the 3 dB coupler to the microscope, the fiber is completely retracted from the cantilever (*d* ≈ 40 mm) so that only a negligible amount of light reflected from the cantilever is collected by the fiber. The light with power *P*_inc_ incident on the fiber end is split into a fraction of power *P*_exit_ exiting the fiber and a fraction of light with power *P*_ref_ that is reflected back inside the fiber (see Figure 2) forming the reference beam for Michelson interference. As *P*_inc_ cannot be measured directly, we introduce the loss factor *f*_loss_ = *P*_inc_/*P* describing the optical loss in the fiber on the way from the beam splitter to the fiber end. The power *P*_exit_ is measured with the power meter after the fiber is cleaved but before it is glued into the ferrule. The back-reflected light with power *P*_ref_ attenuated in the fiber by the factor *f*_loss_ and measured as power 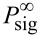 at port 4 of the 3 dB coupler. In this configuration, we find as the relation between the three measured power values:

[3]
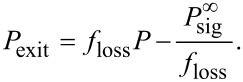


By visual inspection, we find that the amount of light scattered at the fiber end is negligible. Because light absorption at the fiber end can also be neglected, it is straightforward to determine *f*_loss_ = 0.44 ± 0.02 for the experiments reported here. The high loss is presumably occurring in the tightly wound reserve coil inside the vacuum, containing about 3 m of fiber for cleaving new fiber ends and for repairs.

Using this result, we calculate the reflectivity of a fiber end as

[4]
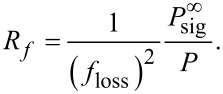


After the determination of the properties of the fiber, *d* is decreased to a distance of approx. 30 μm, estimated through observation with a video camera. The distance is further decreased in single steps until the fiber end is in contact with the cantilever. During the approach, *P*_sig_ is monitored and exhibits interference oscillations. Fiber–cantilever contact is reached when *P*_sig_ does not vary over several steps of approach. Note, that an actual contact between the fiber core and the cantilever is very unlikely, because much more likely, the 125 μm thick fiber cladding surrounding the core will be in contact with the cantilever due to a small unavoidable inclination between the cleavage face and the cantilever. Afterwards, the fiber is retracted by 5–10 μm to protect it against hitting the cantilever during lateral positioning. At this distance, interferometric patterns are observed, which are generated by light with the intensity *I*_inc_ − *I*_ref_ exiting the fiber and entering the interferometric cavity. Inside the cavity, light is reflected back from the cantilever and the fiber-end yielding multi-beam interference inside the cavity where *I*_cav_ is the intensity of the light acting on the cantilever. The actual value of *I*_cav_ depends on the optical losses in the cavity and cannot be determined directly [8].

Part of the light in the cavity is coupled back into the fiber core where it interferes with *I*_ref_ to form the intensity in the signal arm *I*_sig_. The diameter of the fiber core is about five times the wavelength of the light resulting in a light intensity distribution dominated by pinhole diffraction and a diffraction-limited aperture opening angle of 9°. This ensures that the measured values for the optical power represent the respective intensities. The corresponding power value *P*_sig_ is measured by the power meter that can be read out via an analog monitoring port. For dynamic measurements, the intensity is converted into a proportional voltage signal *V*_sig_ via either a custom-built detector-diode/pre-amplifier combination for low-frequency signals or a balanced photo detector (Nirvana detector Model 2007, Newport Corporation, Irvine, USA) processing *P*_sig_ at the signal input and *P* at the reference input.

**Figure 3:**
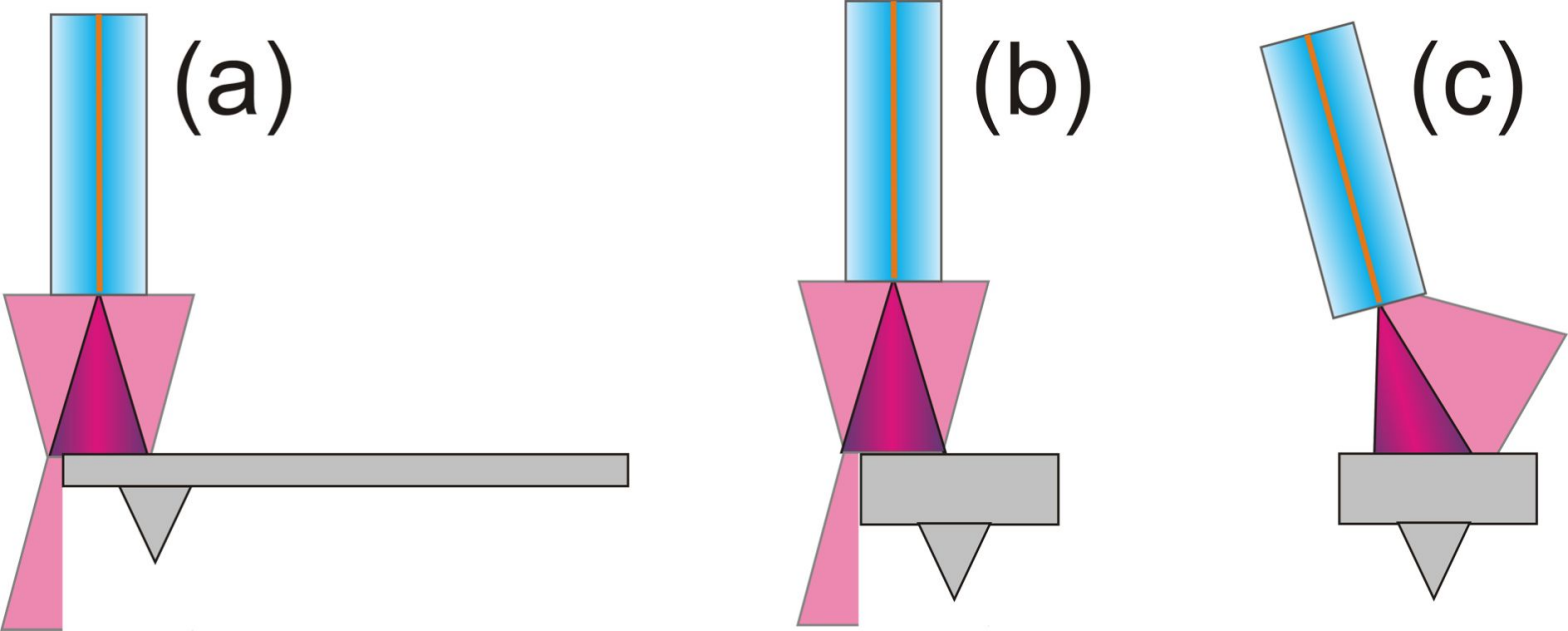
Schematic representation of three common types of misalignment of fiber and cantilever; (a) lateral in length, (b) to the side and (c) tilt of the fiber with respect to the cantilever.

After the approach, the fiber is aligned into the optimal lateral position that is the position of maximum interference signal. For a precise alignment, the lateral cantilever position as well as the alignment angle are crucial. Three types of misalignment resulting in excessive optical loss are shown in Figure 3. A deviation to the cantilever long (Figure 3a) and short (Figure 3b) side can be compensated by adjustment in the *xy*-plane with the fiber piezo. Tilt as shown in Figure 3c can not be compensated by the fiber piezo, but is of minor concern for tilt angles below 4° because of the divergent nature of the beam.

Lateral positioning of the fiber with the tube piezo for alignment and scanning is inevitably accompanied by a tilt of the fiber. The 54 mm long piezo/fiber assembly can be displaced by a maximum of ±10 μm resulting in a maximum tilt angle of 38” and a maximum variation of the distance between fiber end and cantilever of *d* = 4.6 Å. Therefore, we can exclude that the tilt of the fiber changes the interference pattern significantly.

**Figure 4:**
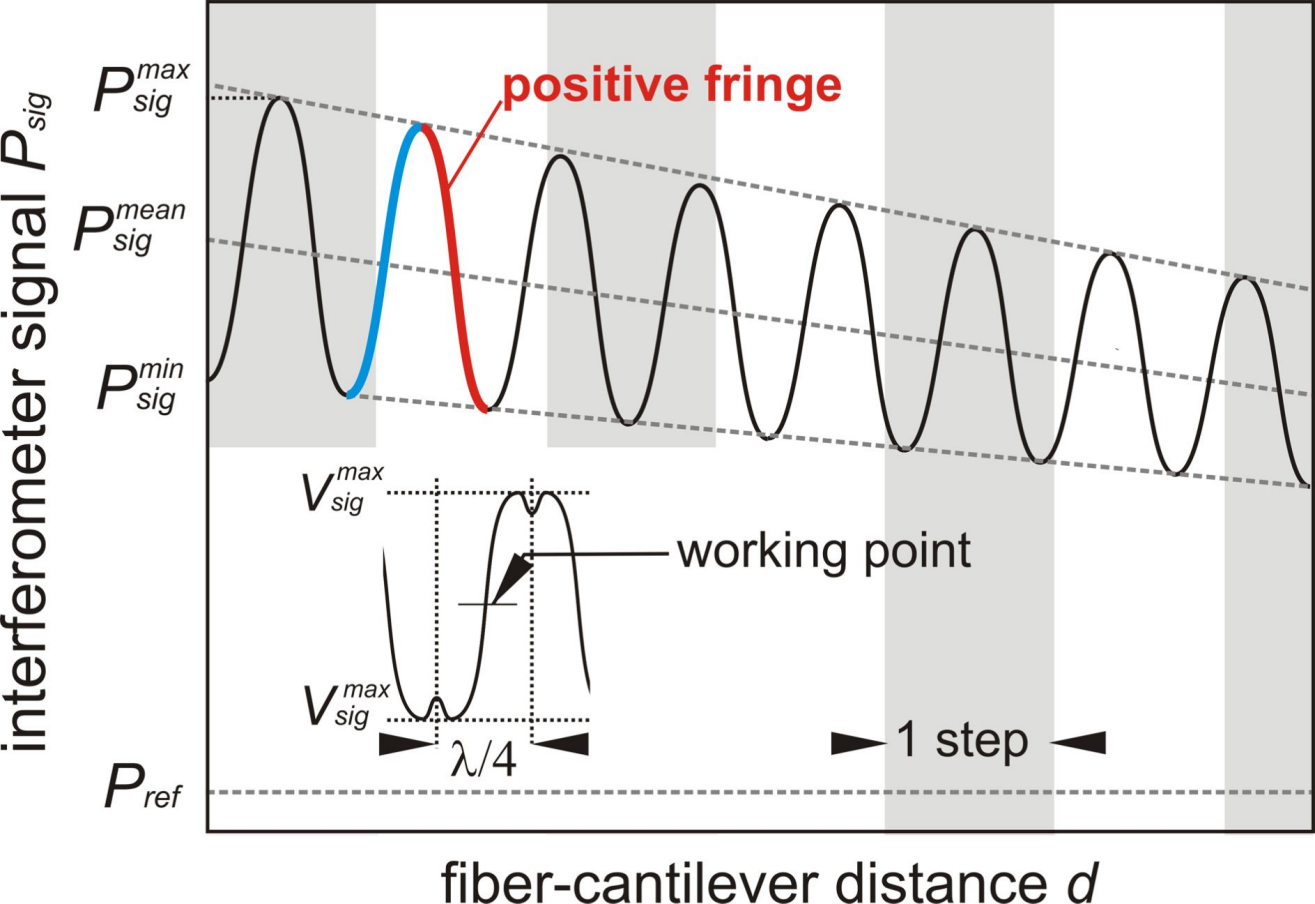
Schematic representation of the interferometer signal *P*_sig_ as a function of the fiber–cantilever distance *d*. Coarse-approach piezo steps are marked as gray boxes. The inset schematically shows the oscilloscope trace of *V*_sig_ for a cantilever excited to an amplitude of larger than λ/8.

Figure 4 is a sketch of the first few interferometric fringes obtained upon retracting the fiber end from the contact position. Practically, only a few fringes can be scanned, limited by the maximum extension of the scanner tube. Starting from the contact point with unknown minimum distance, the signal power varies approximately sinusoidally as a function of *d* between local maxima 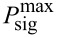 and minima 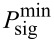 with a distance of λ/2. The optical loss increases with *d* and results in a decrease of the mean value 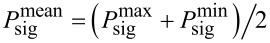 but also of the visibility 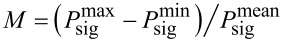, thus being a measure for the Fabry–Pérot enhancement factor 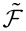
[8].

We define positive and negative fringes so that a positive fringe covers a region of rising light intensity when the fiber is approached towards the cantilever. This is compatible with the more general definition that, for a positive fringe, the force gradient experienced by the cantilever in the cavity light field due to the opto-mechanical coupling points in the same direction as the radiation pressure.

In dynamic operation, the cantilever is excited to oscillation with an amplitude of typically 5–20 nm. To yield the maximum deflection signal, *d* is adjusted so that the zero-crossing of the periodic cantilever displacement coincides with the point of maximum slope for the selected fringe. To find this optimum working point (see inset in Figure 4), the *z*-extension of the fiber piezo is modulated with a frequency of 30 Hz and an amplitude of about 120 nm ≥ λ/8, while *V*_sig_ is observed with an oscilloscope similar to procedures suggested in [13]. The modulation frequency is chosen to avoid mechanical resonances and piezo creep. Dips appear at positions of maximum and minimum *V*_sig_ as schematically illustrated in the inset of Figure 4, as the oscillation extends into neighbouring fringes. If the interference pattern is found not to be symmetric, the *z*-piezo offset voltage is adjusted such that the two dips appear symmetrically. In that way, minimum and maximum voltage levels precisely define the voltage amplitude 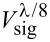 corresponding to a cantilever oscillation amplitude of λ/8. The measured maximum voltage 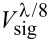 in combination with the wavelength is used for amplitude calibration by applying an arcsine function to account for the approximately sinusoidal modulation of the interferometric fringes as a function of *d*. For a cavity with low 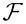, this is a good approximation. Any amplitude *A* below *λ/8* can be determined via

[5]
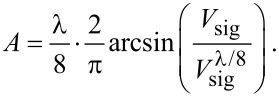


or the commonly used approximation for 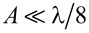.

[6]
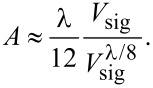


To fine-tune the working point under measurement conditions where the fiber position is fixed, the cantilever oscillation amplitude is adjusted to oscillation with an amplitude of about 10 nm where *V*_sig_ is a quasi-linear function of *d* and the *z*-piezo offset voltage is adjusted such that the maximum peak-to-peak voltage is obtained. Such fine-tuning can be carefully repeated during a series of measurements to compensate for thermal drift. Note, that the measured amplitude corresponds to the position of the light spot on the cantilever, that may differ from the tip position that is relevant for NC-AFM measurements.

## Results

### Cantilever alignment

Signal quality crucially depends on the relative alignment of optical fiber and cantilever, which is in first place determined by the precision in gluing the cantilever support chip on the cantilever holder. Misalignment of the types illustrated in Figure 3 can only partially be corrected by positioning the fiber with the fiber piezo, however, the quality of the cantilever alignment can easily be checked by measuring the signal power *P*_sig_ as a function of *d* revealing the optical loss of the cavity [8]. For such a measurement, the fiber is first positioned in contact with the cantilever and then *d* is increased over a large range via the coarse-positioning stepper in increments of 0.4 μm. Note, that these steps are much larger than one interferometric fringe leading to an aliasing of the interference signal.

**Figure 5:**
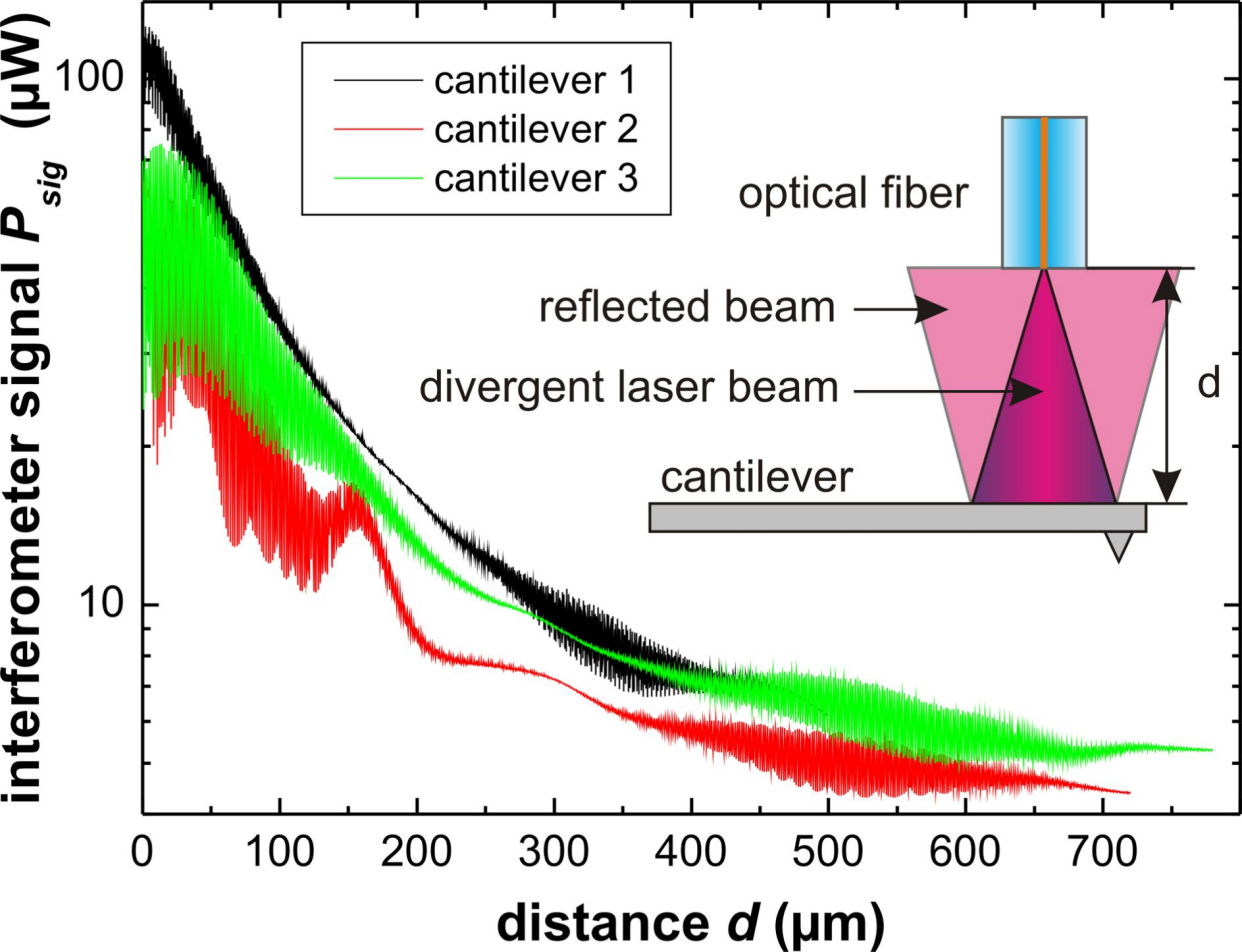
Signal power over distance measurements for three differently positioned cantilevers. Cantilever 1 has high optical loss, cantilever 2 is positioned to the side of the fiber, cantilever 3 is positioned close to the optimum.

In Figure 5, respective measurements are shown for cantilevers 1, 2 and 3 exhibiting different alignment quality. Generally, the increasing optical loss results in an overall decline of the signal when increasing *d* and the signal power *P*_sig_ asymptotically approaches *P*_ref_. For all three cantilevers, two characteristic regimes of interference are visible. At small distances with low cavity loss, Fabry–Pérot interference dominates the signal while for larger distances, Michelson interference dominates the signal. In the distance region between these regimes where no modulation is visible, the interferometric signal is effectively quenched as the light beams originating from Fabry–Pérot and Michelson interference have a similar amplitude but 180° phase shift. As a result of the multi-beam interference, the signal visibility *M* in the Fabry–Pérot regime is up to 14 times larger than that in the Michelson regime.

Cantilever 1 (black curve) exhibits a high overall signal strength, however, the modulation depth is low. This means that the light is reflected back from the cantilever but is lost for the next cavity round trip. This may be caused by the fiber being positioned at the tip of the cantilever as indicated in Figure 3a in combination with a large tilt shown in Figure 3c. Because such a configuration results in a high cavity loss for multiple-beam interference, although a large amount of light reflected back from the cantilever surface enters the fiber. This is in line with the early onset of Michelson interference observed for this cantilever since the high loss results in a strong reduction of multi-beam interference. Cantilever 2 (red curve) is hit close to the side as schematically shown in Figure 3b, effectively decreasing the mirror area for close distances and thus the amount of light reflected back. This results in erratic and low performance of the interference under Fabry–Pérot conditions. Because of the beam divergence, the cantilever is performing better for distances above 300 μm when the entire cantilever is hit by the light resulting in a good signal strength and modulation depth for Michelson interference. Cantilever 3 (green curve) demonstrates close to optimum alignment as the modulation depth is large in both modes of operation. The non-monotonous slope of 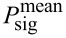 around *d* = 300 μm, however, points to a slight misalignment also for this cantilever. All three examined cantilevers show similar performance in Michelson interference and are equally suited for usage in this regime. However, only cantilever 3 exhibits a performance suitable for further experiments in the Fabry–Pérot regime.

### Fiber positioning

For a perfectly aligned fiber and an infinitely extended mirror surface, there should be no signal variation when scanning the fiber parallel to the mirror surface, as the fiber is the source and the collector of the light. The pattern is expected to exhibit variations with a period of λ/2 upon a variation in *d*. However, the limited area of the cantilever mirror as well as an unavoidable misalignment result in variations for movement parallel to the cantilever surface and, therefore, one can search for the optimal lateral position with minimal optical loss and minimal phase difference between the cavity light beam and the reference beam reflected back inside the fiber.

**Figure 6:**
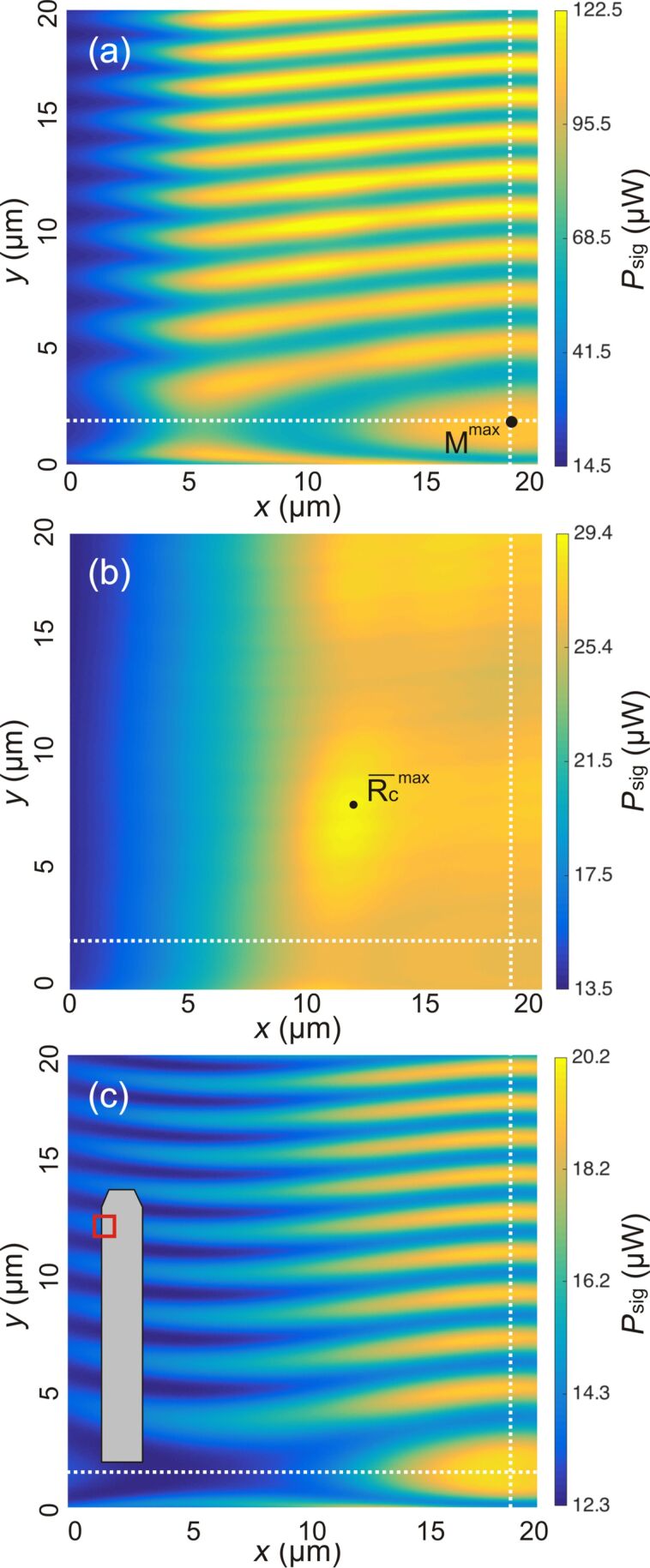
Lateral interference patterns for cantilever 4 scanned by the fiber-positioning piezo for (a) Fabry–Pérot interference *d* = 25 μm, (b) interferometric quenching at *d* = 300 μm and (c) Michelson interference at *d* = 500 μm. The inset shows a sketch of the scanned area in relation to the cantilever. For a full account of the interference pattern, see Supporting Information File 1.

To study alignment effects, the interferometer signal is recorded while laterally scanning the fiber over an area of 20 μm × 20 μm for a fixed *z*. Such patterns are recorded for 512 equidistant slices with *z* ranging from 0 to 5 μm generating a 3D intensity map. From the 3D data, it is straightforward to extract a profile of the interferometric pattern in any plane (see Supporting Information File 1). Results from respective experiments performed with cantilever 4 are shown in Figure 6. The interference pattern for a scan in the *xy*-plane is recorded for (a) Fabry–Pérot interference at *d* = 25 μm, (b) interferometric quenching at *d* = 300 μm and (c) Michelson interference at *d* = 500 μm.

Apparently, there is a signal variation in all images representing different effects. The left side appears darker in all intensity maps, which is the result of a loss of light due to cantilever misalignment as shown in Figure 3b. The most prominent features are, however, the lateral stripes appearing in Figure 6a and Figure 6c. These are a result of *d* changing by scanning along the *y*-axis, which is inclined with respect to the the *y*’-axis as illustrated in Figure 1. Scanning a distance Δ*y* along the *y*-axis results in a distance change of

[7]
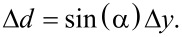


For the fiber positioned close to the cantilever, the interference is dominated by Fabry–Pérot interference represented by Figure 6a. The intensity map is basically a stripe pattern where stripes are aligned parallel to the *x*-axis, as movement parallel to this axis does not change *d*. The small deviation from the alignment is due to a slight misalignment of the cantilever or the fiber. Overall, the stripe pattern is rather even. However, we identify one distinct point of maximum fringe visibility *M*^max^ at *x* = 19 μm and *y* = 2 μm. For Michelson-dominated interference represented by Figure 6c, the resulting pattern is similar to that for Fabry–Pérot interference with *M*^max^ found at the same lateral position. In contrast to the Fabry–Pérot case, here the pattern continues into the wider area of low intensity. At this distance more of the divergent beam hits the cantilever producing interference than in the Fabry–Pérot case where the fiber end is much closer to the cantilever. Furthermore, the light collected by the fiber core is integrated over a larger cantilever area compared to the Fabry–Pérot case, resulting in an overall smoother interference pattern.

**Figure 7:**
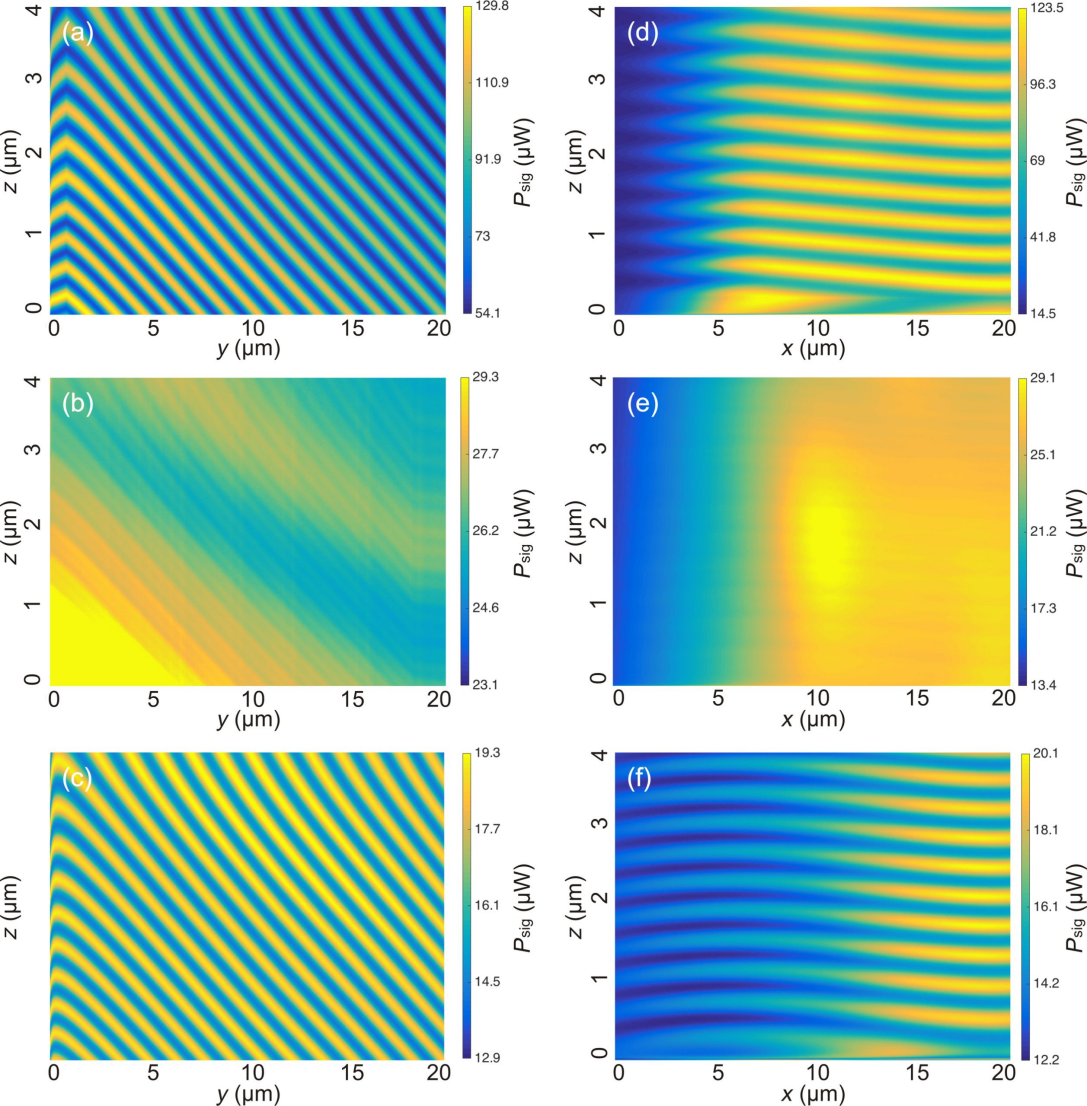
Interference patterns for cantilever 4 generated from the 3D intensity map data for (a,d) Fabry–Pérot interference *d* = 25μm, (b,e) interferometric quenching at *d* = 300 μm and (c,f) Michelson interference at *d* = 500 μm. The *yz*-profiles (a,b,c) where generated for a fixed position *x* = 19 μm, the *xz*-profiles (d,e,f) for a fixed position *y* = 1 μm. For a full account of the interference pattern, see Supporting Information File 1.

The stripe pattern cannot be seen in Figure 6b due to the effective quenching of the interference patterns in the transition regime. Here, the image represents the intensity of the light reflected from the cantilever and the intensity drop at the left side is most pronounced. Note that the overall intensity maximum representing the cantilever reflectivity maximum 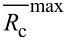 of the cantilever located at *x* = 12 μm and *y* = 7 μm is different from the position of *M*^max^. In a similar fashion, we generate profiles in the *yz*-plane shown with constant *x* = 19 μm in Figure 7a–c and profiles in the *xz*-plane with constant *y* = 2 μm shown in Figure 7d–f. The cuts have been positioned so that both intersect with *M*^max^.

In the *yz*-slices (Figure 7a–c) the diagonal lines represent lines of constant *d* and are inclined by an angle α (see Figure 1) with respect to the *y*-axis. This is utilized to calibrate the sensitivity of the tube piezo in *y*- and *z*-direction. We calibrate by measuring the distance between a local maximum and the (*n* + 1)-th maximum along the *y*- and *z*-directions and use λ as a length standard to obtain the piezo calibration factors *C_y_* and *C_z_*:

[8]
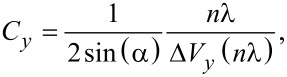


[9]
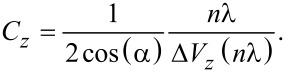


The quantities Δ*V_y_* and Δ*V_z_* denote the difference of voltages applied to the tube piezo corresponding to the positions of the maxima. Analyzing Figure 7a and Figure 7c, we find *C_y_* = 45 ± 4 nm/V and *C_z_* = 10.5 ± 1 nm/V. The calibration in *x*-direction can be assumed to be identical to the one in *y*-direction but can not be measured by this method. At the *y*-position of *M*^max^ at *y* = 2 μm, the direction of the diagonal lines changes. We attribute this to a small deviation of the orthogonal positioning between fiber and cantilever resulting in a position of minimal displacement at Δ*y*’ = 0 and a surrounding pattern with (Δ*y*)^2^ = (Δ*y*’)^2^ + (Δ*d*)^2^.

**Figure 8:**
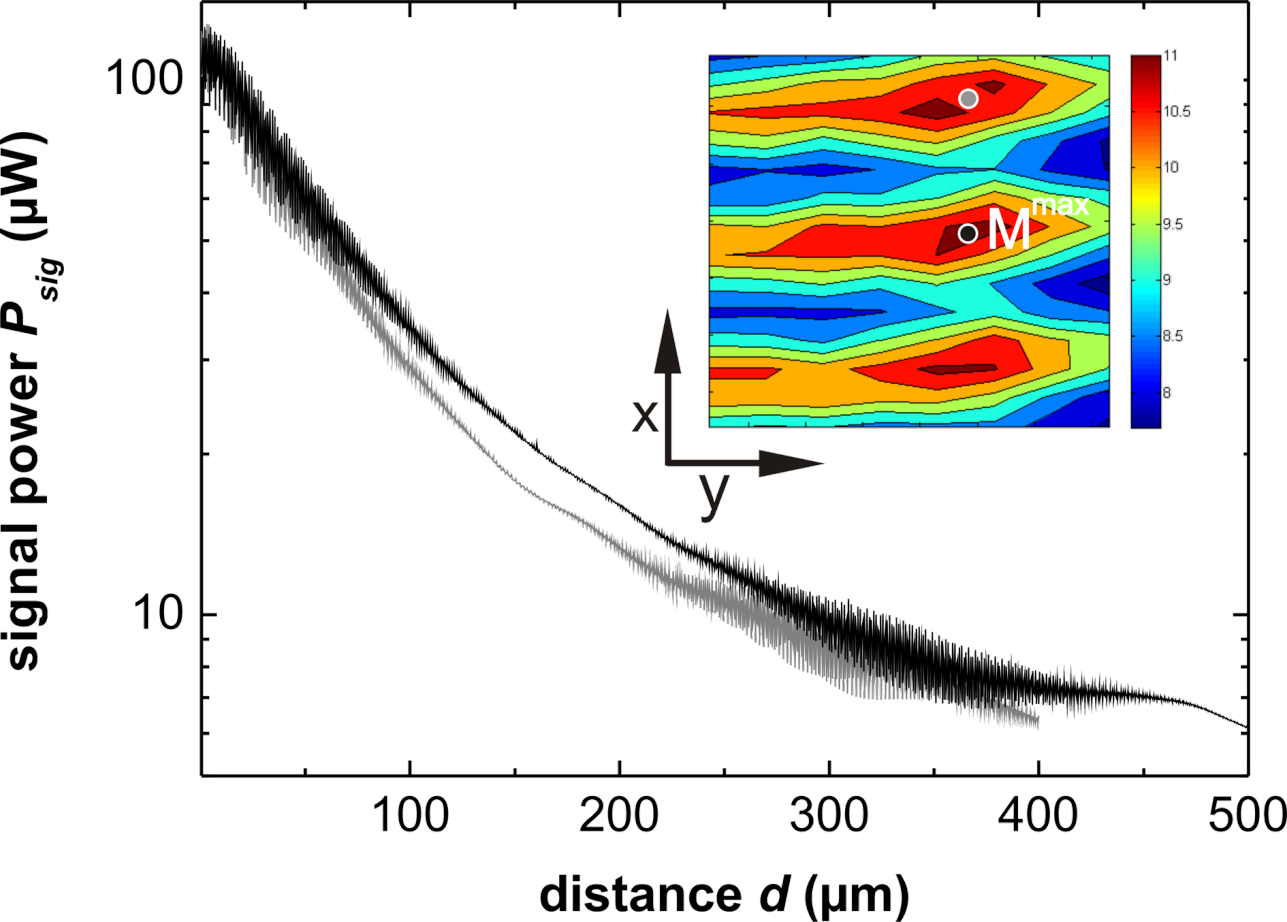
Interferometer signal power *P*_sig_ of the main maximum (black) and a side maximum (gray) measured for cantilever 1 as a function of *d*. The inset shows the pattern in the *xy*-plane around the main maximum for the fiber–cantilever distance *d* = 330 μm, with the positions of the distance-dependent measurements marked with black and gray circles, respectively.

For the slices in the *xz*-plane (Figure 7d–f), we observe almost horizontal lines with the expected sinusoidal signal modulation. The slight tilt of the lines with respect to the horizontal axis is a result of a small cantilever rotation in the *xy*-plane resulting in a movement nominally in *x*-direction having a small component in *y*’-direction. Otherwise, these images can be interpreted as the ones from the other series. The fiber should be positioned for minimum optical loss to maximize the absolute signal strength as well as modulation depth. For cantilever 4, we find this position to be in the Fabry–Pérot mode of operation at *x* = 19 μm, *y* = 2 μm and *z* = 1.7 μm. The lateral position of this point of lowest optical loss is generally found to be the same for all distances, depending only on the cantilever misalignment. To illustrate the impact of lateral positioning on the signal quality, we measure *P*_sig_ over *d* for cantilever 1 analogously to the measurements shown in Figure 5 for the optimum position and a position shifted by Δ*y*’ = λ, respective results are shown in Figure 8 where the positions are marked in the inset. Cantilever 1 is chosen for this purpose since it exhibits the highest optical loss and thus is most sensitive to lateral positioning.

Starting at identical intensities in the Fabry–Pérot region the curves significantly differ from each other for larger distances. However, both curves exhibit identical values for 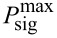 and 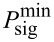 in the maximum of the Michelson mode at *d* = 295 μm and *d* = 340 μm, respectively. This can be explained straightforwardly by an increased optical loss. In the maximum of the Michelson interference, *I*_cav_ collected by the fiber is of the order of *I*_ref_. The increased loss due to the lateral miscalibration is responsible for a faster decrease of *I*_cav_, which directly translates in a compression of the entire interference pattern to smaller *d*. The amount of this compression varies with the cavity loss of the order of 2–12%.

### Cantilever and system noise characterization

For cantilever 3, we investigate the influence of fiber positioning on the effective parameters of the cantilever and the noise performance of the system. Measurements are performed with the balanced detector to yield the best possible noise performance. To characterize the cantilevers and the noise performance of the detection system, we use well-established methods based on the spectral analysis of displacement fluctuations of a thermally excited cantilever [14–15]. For that purpose, the signal spectral density 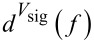 is measured around the eigenfrequency *f*_0_ for the thermally excited cantilever using a HF2 spectral analyzer (Zurich Instruments, Zürich, Switzerland). Results for the effective cantilever parameters are compiled in Table 1. The fringe-dependent effective cantilever stiffness *k*^±^ is determined by a method relating the intrinsic stiffness to the optical spring constant as described in detail in [8].

**Table 1:** Effective modal Q-factor 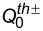, effective modal cantilever stiffness 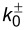 and noise floor 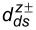 for the positive and negative fringe for 400 μW under conditions of optimal Fabry–Pérot (FP) and Michelson (M) configuration.

*d* [μm]	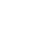	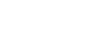	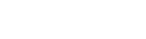

18 (FP +)	18031	55.4	33
18 (FP −)	18925	53.3	44
510 (M +)	17911	54.5	988
510 (M −)	19081	54.4	1065

**Figure 9:**
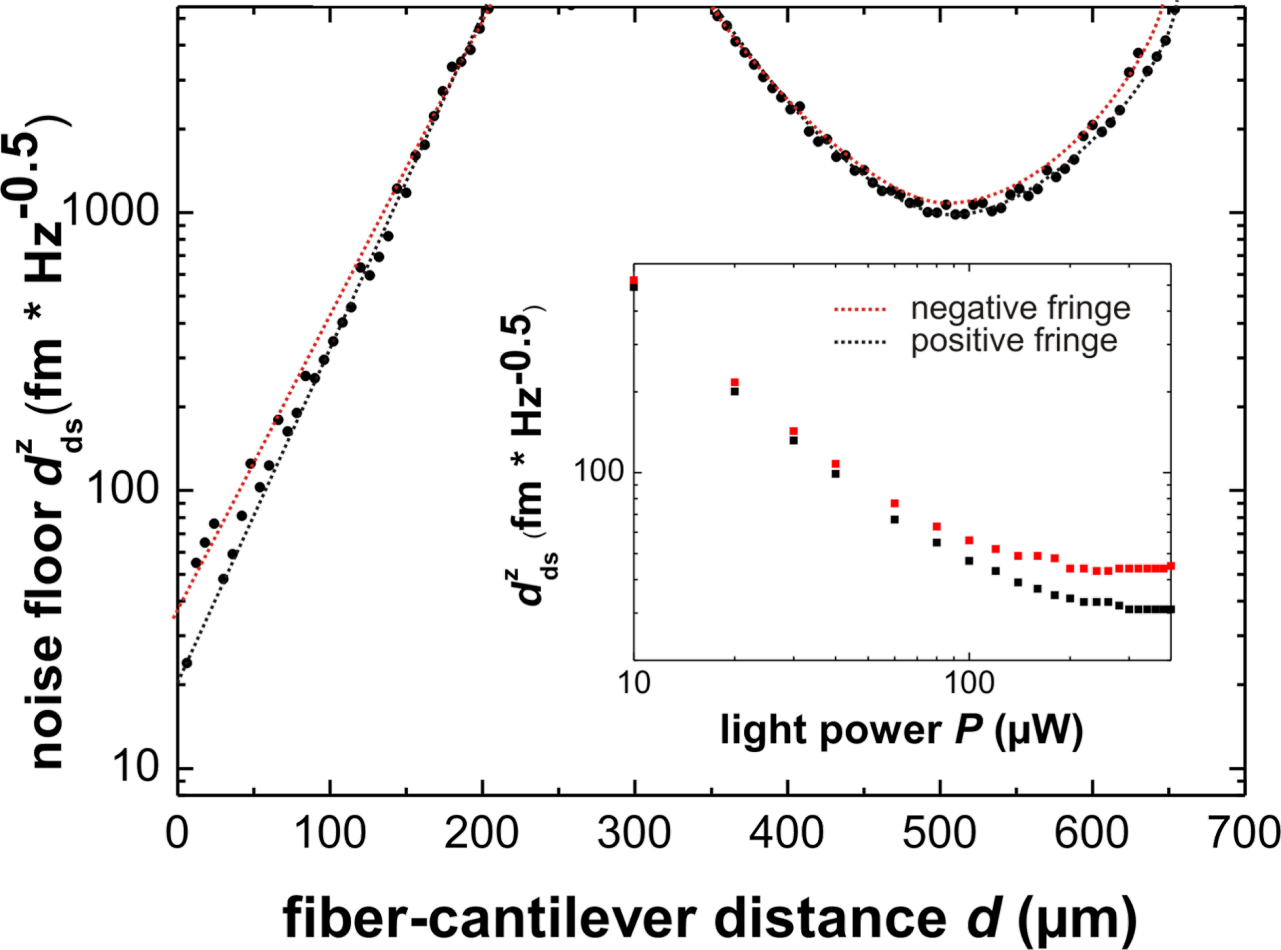
Displacement spectral density 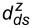 of the noise floor of the interferometer signal as a function of the fiber–cantilever distance *d* for *P* = 400 μW measured for cantilever 3. The inset shows the noise floor for positive and negative fringes at *d* = 18 μm as a function of the light power *P*.

In a series of measurements, we determine the noise floor by measuring the displacement noise spectral density 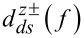 around the eigenfrequency of the fundamental mode *f*_0_ for a laser power of *P* = 400 μW and different values of the fiber–cantilever distance in the range of *d* = 6–660 μm with a step size of 6 μm. The amplitude is calibrated at every position to translate the observed voltage noise spectral density into the displacement noise spectral density 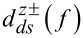. The corresponding results are shown in Figure 9. For *d* = 18 μm, we additionally determine the noise floor as a function of *P* with the results being shown in the inset.

For the Fabry–Pérot regime (*d ≤* 200 μm), we find an exponential increase of the noise level with distance from a minimum equivalent displacement noise spectral density of 
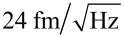
 for *d* = 6 μm to over 
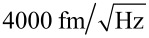
 at 200 μm. Hence, the interferometer exhibits excellent noise figures when operated in the Fabry–Pérot regime with high 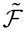.

The dramatic increase of the noise level can be explained by the interference signal *V_sig_* dramatically decreasing with *d* according to the results shown in Figure 5 and Figure 8. For *d ≤* 100 μm, where 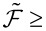 3, we observe a splitting of the curve into two branches corresponding to positive and negative fringes. The splitting is confirmed by measuring the noise floor for *d* = 18 μm as a function of the light power *P* as shown in the inset of Figure 9. The observation of the splitting for *P ≥* 50 μW clearly points to opto-mechanical coupling influencing the stochastic cantilever motion in this regime. The fact that this value is different for neighboring positive and negative fringes with identical values of 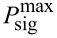 and 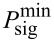 strongly suggests that this limit is not caused by the noise of the laser, the photo diode or following electronics, but is at least partially a result of the opto-mechanical interaction in the cantilever system. Although, the details of this interaction remain to be explored, we find that opto-mechanical coupling is apparently the limiting factor for the noise performance of our system.

In the distance regime between Fabry–Pérot and Michelson operation (200 to 350 μm) the modulation of the interferometer signal is too small to detect a meaningful cantilever oscillation. Above *d* = 350 μm, Michelson interference is dominant and the noise level drops to a value of about 
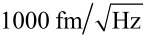
 for the optimum Michelson configuration at *d* = 510 µm. Contrary to the Fabry-Pérot regime, we find that the interferometer is not well suited for low-noise measurements in the Michelson mode in the present configuration of an asymmetric cavity.

To investigate the impact of opto-mechanical coupling on other oscillatory properties of the cantilever, two neighboring fringes with identical values for 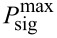 and 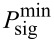 are investigated for the optimal Fabry–Pérot as well the optimal Michelson configurations when determining the effective modal Q-factors 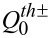 and effective cantilever stiffnesses 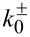 (Table 1) by procedures described in [8,14–15]. The opto-mechanical effects are observable in the cantilever stiffness 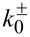 exhibiting the characteristic split between the fringes due to the optical spring effect of up to 4% as expected from our previous studies [8]. For small amplitudes and a negative fringe, the Q-factor is up to 6% larger than for the positive fringe. Although, this variation is significant within the statistical error, it is much less than the typical tolerance of commercial cantilevers and the impact of the mounting system [16]. Overall, we find that the oscillatory cantilever properties are not heavily affected by the interferometric detection while operating the interferometer in the Fabry–Pérot mode with high 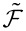.

## Discussion

Interferometric detection is a straightforward and highly sensitive method for measuring displacement in a cantilever-based NC-AFM. This has been realized already in the early days of frequency-modulation force microscopy [2,7,13,17–18]. By instrumental development and optimization, the detection sensitivity has constantly been improved over two decades of development and a force sensitivity in the attonewton range has been claimed for measurements with an ultra-soft cantilever in conjunction with interferometric detection [19]. Although, other variants have been introduced [20–22], the fiber-optic interferometer [23–26] is the most commonly used optical setup for measuring cantilever displacement. This type of interferometer is based on guiding the light entirely through optical fibers and utilizes a 3 dB beam splitter for routing light beams while one cleaved fiber end and the cantilever act as mirrors producing interfering light beams. A ramification of this concept is that light is always delivered and collected through the same aperture defined by the core of the fiber, which typically has a diameter of a few micrometers. Therefore, the vast majority of the sampled light stems from the center of the interference pattern and the challenge in signal detection is just to monitor light intensity variations with the lowest possible noise. The details of interference signal generation can, however, be predetermined by appropriately manipulating the optical reflectivities of fiber end and cantilever with reflective coatings. According to the preference of the experimentalist, signal generation can be based predominantly on two-beam interference of Michelson-type or multi-beam interference of Fabry–Pérot-type, the latter with either low or high finesse. While a Michelson-type interferometer is simple in adjustment and robust in operation [27–28], the high-finesse cavity of a Fabry–Pérot interferometer yields high optical signal amplification but requires a sophisticated cavity design or active stabilization [29–31].

Here, we described an interferometer with a strongly asymmetric low-finesse cavity combining the high reflectivity of a metal-coated cantilever with the low reflectivity of the bare, cleaved fiber end, which is a simple design previously adapted by several authors [17–18,32–33]. In previous work, we have shown that this allows for a smooth transition from predominant Fabry–Pérot operation to predominant Michelson operation by adjusting the gap between the fiber end and the cantilever, effectively controlling the cavity optical loss [8]. For the case of multi-beam interference, the interaction between the cavity-amplified optical field and the cantilever results in opto-mechanical coupling and a shift of several parameters of the cantilever oscillation to fringe-dependent effective values. This allows, for instance, for a simple determination of the effective cantilever stiffness for operation in positive and negative fringes as well as the intrinsic cantilever stiffness. We find that the Fabry–Pérot mode of operation with the smallest fiber–cantilever gap allows for displacement detection with a very low detection-system noise floor of 
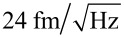
 promising a high sensitivity in force detection even for the relatively stiff cantilevers used in the present study. Apparently, the system noise is affected by opto-mechanical coupling and we find that measuring on a positive fringe is the best choice with regard to noise. However, in future work the opto-mechanical coupling might also be used to advantageously manipulate the cantilever dynamics for improved force detection and measurement stability.

## Supporting Information

File 1 Profile of interferometric patterns in all planes.
